# The consistency approach for the substitution of *in vivo* testing for the quality control of established vaccines: practical considerations and progressive vision

**DOI:** 10.12688/openreseurope.15077.1

**Published:** 2022-10-05

**Authors:** Jean-Francois Dierick, Marlies Halder, Carmen Jungbaeck, Julie Lorenz, Jean-Marie Préaud, Patrice Riou, Lorenzo Tesolin, Sylvie Uhlrich, Wim Van Molle, Joris Vandeputte

**Affiliations:** 1 GSK Vaccines, 89 Rue de l’Institut, Rixensart, 1330, Belgium; 2 Joint Research Centre, European Commission, Via E. Fermi 2749, Ispra (VA), 21027, Italy; 3 IABS-EU, 24 Rue Baldassini, Lyon, 69007, France; 4 Zoetis Inc., 333 Portage St, Kalamazoo, MI, 49007, USA; 5 Sanofi Vaccine, 1541 avenue marcel Mérieux, Marcy l’Etoile, 69280, France; 6 Quality of Vaccines and Blood Products, Sciensano, 14 rue J. Wytsman, Brussels, 1050, Belgium

**Keywords:** consistency testing, vaccines, in vitro, in vivo, validation, analytical method

## Abstract

The aim of this letter is to share the discussions and proposals made by the VAC2VAC consortium on how to support the deployment of the “Consistency Approach” for quality control of established vaccines and thus facilitate the substitution of *in vivo* testing. This work answers specific questions about “ *what does a control strategy according to the consistency testing look like”* and “ *how to submit a control strategy defined according to the consistency testing”*. Some topics were answered in a very straightforward manner. This was the case when the deployment of the consistency approach and the corresponding changes in vaccines control strategy can be supported by the generic application of processes already described in regulatory guidelines/requirements and related to the establishment or change in the control strategy of vaccines. The application of some other processes required specific attention while others were deeply debated before reaching a proposal. The key outcomes of this work are that robust science must be used to develop a substitution strategy and produce supportive data packages. And this good science will be more efficient when supported by scientific collaboration between the different parties involved. Therefore, early interaction between manufacturers and competent authorities before and during dossier submission are key elements to success. The consistency approach, when approved and in place, will ensure vaccine products of assured quality reach the patient in a more efficient manner than when relying on *in vivo* testing. Adapting the mindset was one of the major hurdles to a progressive vision but there is now a consensus between experts from industry and regulatory bodies to foster the elimination of *in vivo* testing for routine vaccine release testing.

## Plain language summary

VAC2VAC is a collaborative research project funded by IMI2, which aims to develop approaches for the testing of vaccines (human or animal) using non-animal methods. In that perspective, the authors launched a reflection on how to support the deployment of the “Consistency Approach” which had been defined in order to facilitate and accelerate the substitution of current *in vivo* methods with *in vitro* alternatives for quality control of vaccines.

 The key outcomes of this work are that robust science must be used to develop a substitution strategy and produce supportive data packages. And, this good science will be more efficient when supported by scientific collaboration between the vaccine manufacturers and competent authorities before and during dossier submission are key elements to success.

## Disclaimer

The views expressed in this article are those of the authors. Publication in Open Research Europe does not imply endorsement of the European Commission. The views expressed by the regulators who have contributed to this article do not *per se* represent the views of the organizations they belong to.

## Introduction

VAC2VAC is a wide-ranging collaborative research project funded by IMI2 (2016–2022), which aims to develop and validate quality testing approaches (physicochemical, immunochemical methods, cell-based and multi-parametric) for routine batch quality, safety and efficacy testing of human and veterinary vaccines using non-animal methods. More information about the VAC2VAC program can be found on the Cordis database from The European Commission: Vaccine lot to Vaccine lot comparison by consistency testing. | VAC2VAC Project | Fact Sheet | H2020 | CORDIS | European Commission (europa.eu). In that perspective, the initiative launched reflection on how to support the deployment of the “Consistency Approach” for quality control of established vaccines, where current quality control approaches are often relying on *in vivo* methods.

Discussions started with experts from industry and regulatory bodies, involved in the VAC2VAC project, on what does a control strategy according to consistency testing entail and how to submit a control strategy defined according to the consistency testing. Then the consortium organized two workshops (on June 22, 2020 and June 24, 2021) regrouping VAC2VAC experts and experts not involved in the VAC2VAC consortium and who represent important stakeholders into the discussions (academics, National Control Laboratories, regulators, governmental and trans-governmental organizations). The goal was to address all topics (simple or complex) that could be related to the change of control strategy and facilitate a common view from regulators and industries in anticipation of future submission of a method variation dossier or a marketing authorization application. Consensus was reached, and proposals were made on several topics. The aim of this document is to share these proposals to a larger audience and stimulate discussion and engagement toward the consistency approach for the substitution of *in vivo* testing for quality control of established vaccines.

This paper summarizes the discussions and proposals made by the VAC2VAC consortium composed of experts, regulators and scientists from different institutions, manufacturers and competent authorities on how to support the deployment of the “Consistency Approach” for quality control of established vaccines. Table 1 summarizes the current regulatory framework taken into consideration during these discussions.

**Table 1.** 

RELEVANT EU-PROVISIONS
**(Veterinary) International Conference on Harmonisation ((V)ICH)**
ICH Q6B ”Specifications: Test Procedures and Acceptance Criteria for Biotechnological/Biological Products” for setting specifications limits.
ICH Q2 (R1) “Validation of analytical procedures”
**European Union**
Directive 2010/63/EU of the European Parliament and of the Council of 22 September 2010 on the protection of animals used for scientific purposes. Official Journal of the European Union L 276, 20.10.2010, p. 33–79. https://eur-lex.europa.eu/eli/dir/2010/63/oj
Communication from the Commission – Guideline on the details of the various categories of variations to the terms of marketing authorisations for medicinal products for human use and veterinary medicinal products https://ec.europa.eu/health/sites/health/files/ files/betterreg/pharmacos/classification_guideline_adopted.pdf
Commission Regulation (EU) No 712/2012 of 3 August 2012 amending Regulation (EC) No 1234/2008 concerning the examination of variations to the terms of marketing authorisations for medicinal products for human use and veterinary medicinal products https://ec.europa.eu/health//sites/health/files/files/eudralex/vol-1/reg_2012_712/reg_2012_712_en.pdf
Commission Regulation (EC) No 1234/2008 of 24 November 2008 concerning the examination of variations to the terms of marketing authorisations for medicinal products for human use and veterinary medicinal products and on the documentation to be submitted pursuant to those procedures https:// eur-lex.europa.eu/LexUriServ/LexUriServ.do?uri=OJ:C:2013:223:FULL:EN:PDF
**European Medicines Agency (EMA)**
Guideline on the principles of regulatory acceptance of 3Rs (replacement, reduction, refinement) testing approaches (EMA/CHMP/ CVMP/JEG-3Rs/450091/2012)
CMDh Recommendation for classification of unforeseen variations according to Article 5 of Commission Regulation (EC) 1234/2008 https://www.hma.eu/fileadmin/dateien/Human_Medicines/CMD_h_/procedural_guidance/Variations/Art_5_Recommendations/CMDh_ 172_2010_09_2019_Tracking_Table_Article_5.xls
CMDv Recommendation for classification of unforeseen variations according to Article 5 of Commission Regulation (EC) 1234/2008 https://www.hma.eu/fileadmin/dateien/Human_Medicines/CMD_h_/procedural_guidance/Variations/Art_5_Recommendations/CMDh_ 172_2010_09_2019_Tracking_Table_Article_5.xls
Reflection paper providing an overview of the current regulatory testing requirements for veterinary medicinal products and opportunities for implementation of the 3Rs (EMA/CHMP/CVMP/3Rs/164002/2016)
Guidance for individual laboratories for transfer of quality control methods validated in collaborative trials with a view to implementing 3Rs (EMA/CHMP/CVMP/3Rs/94436/2014)
Recommendation to MAHs, highlighting the need to ensure compliance with 3Rs methods described in the Ph. Eur. – applicable to all medicinal products regardless of type. (EMA/CHMP/CVMP/JEG-3Rs/252137/2012)
Recommendation to MAHs, highlighting recent measures in the veterinary field to promote 3Rs measures described in the Ph. Eur. – applicable to veterinary vaccines from 01/01/2017.(EMA/CHMP/CVMP/3Rs/336802/2017)
Statement of the CVMP position on the ethical use of animals in the testing, development and manufacture of veterinary medicines (EMA/CVMP/3Rs/506841/2017)
DRAFT Regulation (EU) 2019/6 of the European Parliament and of the Council on veterinary medicinal products and repealing Directive 2001/82/EC
EU-Regulation 2019/6 Draft Annex 2 SECTION IIIb REQUIREMENTS FOR IMMUNOLOGICAL VETERINARY MEDICINAL PRODUCTS IIIb.2D. Control tests during the manufacturing process IIIb.2E. Control tests on the finished product
**European Pharmacopoeia (EP)**
Ph. Eur. general chapter 5.2.14
RELEVANT US-PROVISIONS
USDA Veterinary Services Memorandum No. 800.112, Guidelines for Validation of In Vitro Potency Assays, April 10, 2015.
USDA Veterinary Services Memorandum No. 800.124, Guidelines for Potency Specifications of Biological Products Administered to Animals, October 2, 2020. https://www.usp.org/sites/default/files/usp/document/our-impact/covid-19/standards-for-quality-vaccines.pdf

The use of the consistency approach can replace the current approach for many established vaccines which involves *in vivo* testing known to be laborious and highly variable compared to *in vitro* assays and that are therefore no longer seen as the Golden standard (see Stalpers *et al*., 2021). Robust science and early interaction between manufacturers and competent authorities before and during dossier submission are certainly key elements to success. This consistency approach, when approved and in place, will allow new and established products of assured quality to reach the patient in a more efficient manner.

Science, newly developed methods and relevant guidelines are available to support the move to a Consistency Testing - based - control strategy. Adapting the mindset was one of the major hurdles to a progressive vision but there is now a consensus between experts from industry and regulatory bodies to foster the elimination of *in vivo* testing for routine vaccine release testing.

### The consistency approach

According to de Mattia *et al*. (2011): “ *The consistency approach implies the use of a set of parameters to constitute a product profile (e.g., antigen content, antigen integrity, etc.) that can replace current release tests. The product profile is established to the satisfaction of the regulators at the time of licensing, and is monitored throughout production under a strict quality system. The product profile ensures that each batch or lot released is similar to a manufacturer-specific vaccine of proven clinical efficacy and safety, with respect to all characteristics agreed upon at licensing between manufacturer and regulator.”*

The VAC2VAC consortium proposes the following update of definition from de Mattia *et al*. (2011) to reflect current developments: “ *The consistency approach implies the use of a (set of) parameter(s) to constitute an integrated control strategy preferably relying on non-animal testing (e.g. antigen content, antigen integrity, etc.) and taking into account production controls (e.g. defined ranges, harvest criteria etc.). Taken together it can be seen as an approach which can replace the current approach for many established vaccines which involves in vivo testing. The new integrated control strategy has to be established to the satisfaction of the regulators and is monitored throughout production under a strict quality system. The integrated control strategy ensures that each batch or lot released is similar to a manufacturer-specific vaccine of proven clinical efficacy and safety, with respect to all characteristics agreed upon at licensing between manufacturer and regulator.*

*If available, comparison to the batches used in the clinical studies / challenge studies / field trials to support the marketing authorization application may be an option. For older vaccines, parallel testing of batches may be the better way. The number of batches to be tested in parallel should be reasonable, depending on the number of batches normally manufactured in one year (blockbuster versus orphan products / MUMS).”*

## Proposals on the use of the consistency approach

The following paragraphs describe the proposals emerging from the work from the VAC2VAC consortium on the use of the consistency approach for the substitution of *in vivo* testing for the quality control of established vaccines.

### The simple straightforward topics

Some very simple straightforward conclusions will be shared in this first part in order to express clearly what does not need to be changed in order to apply the consistency approach.

First, it is acknowledged that a Consistency Testing - based - control strategy can be defined using **currently existing concepts and terms** (Critical Quality Attribute, In Process Control, release assays, characterization assays, monitoring assays, …).

For instance, the Critical Quality Attribute (CQA) “potency/safety” might be replaced by several CQAs that are that are relevant for the biological activity/safety of a given vaccine. Process Control might be considered as part of the Consistency Testing - based - control strategy if relevant and necessary in combination with Critical Quality Attributes for the Drug Product (final product).

“Relevant In Process Controls” refer to control tests that have been shown to ensure that a given component of the Critical Quality Attribute “potency/safety” (example: conformation) is maintained when the Critical Process Parameter (CPP) is met throughout the production step.

In all these cases, currently existing concepts and terms are sufficient to define and clearly explain a control strategy that is based on the Consistency Approach.

**Some common processes and practices are also fully applicable** without any specific attention: Analytical Method Validation and Certificates of Analysis. The current provisions on validation should be applied as required under the current control strategy. Results derived with new methods must be included in the Certificate of Analysis when applying (or not) the Consistency Approach. Critical Process Parameters used in the context of Consistency Testing should not be included in the Certificate of Analysis (i.e., no change compared to the current situation). In Process Control assays might be used for release on a Consistency Testing - based - control strategy (for instance, when they cannot be performed at the final stage due to product/matrix changes). In that case, In Process Control results may appear on the Certificate of Analysis.

Finally, could new method(s) developed in the context of the deployment of a Consistency Testing-based - control strategy become compendial? Any new method, e.g., for potency testing, can become compendial (e.g., become part of a monograph from the European Pharmacopeia, initiated by a request for revision). It is understood that new technologies/methods should ideally be platform technologies and/or product group specific and/or product or manufacturer specific. The rationale for a method to become compendial is for harmonization purposes. An international reference can be developed, and a monograph can be edited using common units and specifications. It is important to note that having a method recognized as compendial will promote its usage. Therefore, the introduction of methods that are alternatives to *in vivo* methods in the compendia is an important lever towards the reduction of animal use in vaccine quality control.

Some **other** **common processes and practices require specific attention** and are presented hereunder.

### Product characterization package

When shifting to a Consistency Testing - based - control strategy, the characterization package that will be deployed to assess process changes should be defined and endorsed by a competent authority. It may contain new *in vitro* characterization assays but no *in vivo* assay(s). Indeed, when approved by competent authorities as part of the registration dossier, new methods are considered to be in force. Substituted *in vivo* methods should no longer be used and therefore should not be requested in case of process change.

### Acceptance criteria

Acceptance criteria (or other types of limits) will be set for the new assay(s) that will substitute *in vivo* potency/safety tests. The VAC2VAC proposal highlights that there is no need to change the way acceptance criteria are defined for the new assay(s) that will substitute *in vivo* potency/safety tests. When properly defined, they are reflecting the variability of the (new) method(s) combined with the normal variability of the manufacturing process. It is important to insist on the fact that the introduction of a new analytical method neither changes the variability of the manufacturing process nor the quality of the product.

It is also important to acknowledge that, in the current control strategy, the Critical Quality Attribute (CQA) “potency” is addressed by an *in vivo* test. These tests have an inherently high variability with a range difficult to quantify. Setting specifications for the new in *vitro* methods may therefore be challenging when seeking correlation with the specification established for the *in vivo* assay. This correlation might even be impossible to establish in case the units used in the *in vivo* and the *in vitro* assay are different. Moreover, head-to-head comparison to clinical batches used to demonstrate efficacy/safety may not be possible for already licensed vaccines, since these batches will no longer be available or may be expired.

Taking these elements into consideration, the acceptance criteria of proposed *in vitro* tests will, in most cases, be set on collection of data from released batches – in order to maintain consistency of batch quality – rather than through seeking correlation to acceptance criteria established for the *in vivo* test. Data collection for setting the acceptance criteria must be performed in representative conditions of the future batch release (e.g., if the product is demonstrated to be stable, batches of different ages can be tested retrospectively with the *in vitro* assay. If the product is known to change over time, batches must be tested at the time of release with the *in vitro* assay in order to acquire representative data).

This should be supported by data demonstrating the ability of the proposed assay(s) to control key quality attributes of the vaccine and maintain the link between the quality of the batches to be released using the proposed Consistency Testing - based - control strategy with those batches released as “compliant” when tested with the *in vivo* - based - control strategy. Those latter “compliant” batches having been consistently demonstrated to present the same quality as the clinical batches which have been demonstrated safe and efficacious through clinical studies.

The release and stability specification and acceptance criteria for the new method(s) have to be set on a sufficient number of batches, also taking into account variability of the new method(s). Only limited information will be available on the variability between batches for the new assay at the time of implementation because only a few batches will have been tested with the new assay. When only limited batch results for the new method are available the acceptance criteria may initially be set somewhat wider to account for potential inter-batch variability. A predicted revision of the release specification acceptance criteria after sufficient batches have been tested could be part of the implementation strategy. 

This proposal is supported by the following elements. The quality of vaccines is determined by their design, development, in-process controls, release controls, and process validation, and by specifications applied to them throughout development and manufacture. Specifications, i.e., those tests, validated procedures, and acceptance criteria play a major role in assuring the quality of the product and intermediates at release and during shelf life. Acceptance criteria need to be set in line with the various guidance issued by e.g., text from the European Pharmacopeia issued by the European Directorate for the Quality of Medicines & HealthCare and other legal documents of relevance issued by other competent authorities outside the EU wherever this approach will be accepted.

Recently produced batches can be considered comparable to the original clinical batches, because they are also demonstrated by testing against bridged reference material equivalent to original clinical batches (e.g., Diphtheria and Tetanus) and/or are manufactured by a process demonstrated to be comparable, in cases where manufacturing changes have been introduced.

### Stability studies

*In vivo* potency tests are often used in stability studies. Given the potential configuration of the Consistency Testing - based - control strategy it is important to consider how stability studies should be managed after the removal of *in vivo* potency.

Stability studies per se, do not need to be managed in a different way. After removal of the *in vivo* assay, results generated with the new method(s) being indicative for stability can be accepted, provided that they are within approved specifications throughout product shelf-life.

The strategy for stability testing needs to be adapted to the new control strategy and according to the respective stability-indicating capacity of the new assays as well as the process steps where they are performed. For instance, in a scenario where an *in vivo* potency assay is replaced by an assay measuring the antigen content and another assay assessing the structural integrity of the antigen, the assay for content will not be introduced in the stability plans because it is likely not stability indicating. The structural assay, being demonstrated to be stability indicating could be introduced in stability plans if the stability of the antigen requires evaluation according to current product stability knowledge.

The definition of the acceptance criteria should take into account stability data. If the results generated with the *in vivo* substitution method(s) show a decrease/increase over time, an end-of-shelf-life acceptance criterion can be defined for the vaccine provided that it is not in contradiction to current regulatory requirements and that it is in line with principles of e.g., ICH Q6B “Specifications: Test Procedures and Acceptance Criteria for Biotechnological/Biological Products”.

*In vivo* assays used in stability studies generally have a different discriminative power than *in vitro* assays regarding the various effects that may impact the product along stability. Therefore, the strategy for stability testing needs to be adapted to the new control strategy.

Change of the shelf-life criteria during storage can partly be assessed by accelerated stability studies but can only be confirmed in real-time stability studies. Final stability criteria have to be based on batches at the end of shelf-life. Again, a foreseen revision of end of shelf-life acceptance criteria may be part of the implementation strategy.

### The role of Official Medicines Control Laboratories (OMCLs)

Technically all assays can be performed by Official Medicines Control Laboratories (OMCLs) regardless at the production step they are performed and this does not change with a control strategy based on the Consistency Approach.

Providing samples either of the final lot or from upstream process steps is required in the regulation of official batch release procedures. An upstream sample might need to be sent to the control laboratories before the final lot submission to gain time for release testing and to respect testing within shelf-life.

In Europe, after approval of the data package submitted to EMA, the list of tests to be performed is defined by the EDQM - OCABR drafting groups and approved by consultation of the OMCL network.

In the European batch release framework (as laid down in Council Directive 2001/83/EC, Article 114 for medicinal products for human use and in Regulation 2019/6/EU Art 128 for medicinal products for veterinary use), an Official Medicines Control Laboratory (OMCL) tests a biological product before it can be marketed. The list of tests to be performed is prepared by the OCABR drafting group of experts addressing product quality and approved by consultation of the relevant EDQM OMCL network. Tests are chosen from those approved in the data package submitted to EMA. For example, the potency test is part of product quality evaluation package and, therefore, any tests replacing the potency test should be performed by the OMCLs. By extension, National Control Laboratories outside Europe could also perform the new assay(s) for the product release in their market or rely on the European batch release certificate.

## Additional key elements to success

The keys to success are in the scientific approach combined with early interactions between manufacturers and regulatory authorities.

Even beyond the development of fit-for-purpose, alternative *in vitro* assays, the topic that triggered most discussions in the VAC2VAC consortium is how to build data packages that demonstrate that the change towards the new proposed Consistency Testing - based - control strategy can be achieved. The answer to that challenge is composed of three elements: including scientific relevance of the proposed *in vitro* assays, the use of non-compliant batches in the assessment of the control strategy and early interactions between manufacturers and authorities to discuss the proposed change(s). This approach reflects the fact that applying good science is the best way to move forward on such complex and sometimes sensitive topics. And this good science will be made more efficient when supported by scientific collaboration between the different parties involved (and not simply scientific communication).

### The science: the use of non-compliant batches

Demonstration of agreement between *in vitro* and *in vivo* methods may not be scientifically justified and should not always be expected (Ph.Eur. 5.2.14 Substitution of *in vivo* method(s) by *in vitro* method(s) for the quality control of vaccines). Batches that differ from compliant batches (e.g., partially degraded, lower content, impurities) tested by *in vivo* tests in terms of quality should be used to demonstrate that the *in vitro* assay is fit-for-purpose.

The use of non-compliant batches is important for the demonstration of the ability of the new assay(s) to discriminate between compliant and non-compliant batches when changing from an existing *in vivo* - based - control strategy to a proposed Consistency Testing - based - control strategy.

However, the comparison of results obtained on these non-compliant batches using an *in vivo* method and the proposed *in vitro* method(s) is neither considered as the best nor as a sufficient approach. Indeed, Ph.Eur. 5.2.14 states the following “…a demonstration of agreement between the 2 methods is generally not scientifically justified and should not always be expected. Even where pass/fail results from the 2 test procedures are in agreement, the correlation between 2 quantitative methods across the assay range may still be low. Regardless, the *in vitro* method(s) or testing strategy must provide at least the same confidence that the key quality attributes, which are necessary to ensure the consistency of a product’s safety and effectiveness, are adequately controlled.”

The reasons supporting that statement are:

- *In vivo* methods present a high level of variability that do not allow proper, statistically- relevant comparison of methods based on a reasonable amount of data (and animals).

- Historically, *in vivo* methods were generally not designed to address a well-defined Critical Quality Attribute but mostly rely on the belief that the product will trigger the same overall response in test animals as in target species.

- Modern analytical technologies allow the building of more relevant and comprehensive data sets about product characteristics/knowledge than *in vivo* assays.

Therefore, the demonstration that changing from an existing *in vivo*-based-control strategy to a proposed Consistency Testing - based - control strategy must rely on data/evidence that the proposed Consistency Testing - based - control strategy can ensure the control of well-defined Critical Quality Attribute(s) that are contributing directly or indirectly to the quality attributes being supposedly measured by the *in vivo* assay(s) to be substituted. This consists in the demonstration that the level of control of the proposed Consistency Testing - based - control strategy is appropriate in order to provide consistently a product of the desired quality.

### Early scientific interactions between manufacturers and authorities

Manufacturers and regulator(s) must agree on an overall strategy and the type of data to be provided in a future variation/licensing application. A scientific discussion with competent authorities is of utmost importance and should start as early as possible. It is necessary to define different data/justification packages recommended for proposing *in vivo* potency substitution for different assays/individual products. It is recommended that specific data/justification packages are defined for proposing *in vivo* potency/safety test substitution of the various assays/individual products.

The various guidance documents on how to apply for scientific advice issued by the competent authorities should be followed.

The manufacturer is advised to have an early scientific discussion with competent authorities.

These discussions can address multiple levels of complexity:

- Rational explaining the scientific relevance and the added value of the proposed control strategy
- Outline of the proposed control strategy (new assays to be introduced, usage of these assays, e.g. stability)
- Proposed plan to generate the data package to support the change in control strategy

The data/justification package needs to be approved by competent agencies on a case-by-case basis. The approach should be in line with Ph.Eur. Chapter 5.2.14.: “The test methods used for routine quality control of vaccines are intended to monitor production consistency and to ensure comparability of the quality attributes between commercial batches and those batches originally found to be safe and efficacious in clinical studies, or for veterinary vaccines, in the target species.”

At any stage of the method development and validation, the scientific discussion with the competent authorities helps the manufacturer to develop the appropriate strategies, tests including specification limits and studies, so that major objections regarding the design of the *in vivo* substitution tests are most unlikely to be raised during the evaluation of the variation/licensing data package. The discussions can be very useful in light of the complexity of this type of change in the control strategy.

The data/justification packages should be elaborated based on research and development studies, historical results generated on manufactured batches, stability studies, and non-conforming batch studies, in accordance with the advice given by regulatory agencies during the staggered approach and in line with the different guidelines in force. They can differ from one case to another due to the following: potency vs safety test, one or more *in vitro* test(s) to substitute an *in vivo* test, drug substance vs drug product stage, etc. Those data packages should therefore be considered on a case-by-case basis.

The discussions between manufacturers and regulators are thus critically important in light of the complexity of this type of change in the control strategy.

## Conclusions

 This paper summarizes the discussions and proposals made by the VAC2VAC consortium composed of experts, regulators and scientists from different institutions, manufacturers and competent authorities on how to support the deployment of the “Consistency Approach” for quality control of established vaccines. The use of the consistency approach can replace the current approach for many established vaccines which involves *in vivo* testing known to be laborious and highly variable compared to *in vitro* assays and that are therefore no longer seen as the Golden standard. Robust science and early interaction between manufacturers and competent authorities before and during dossier submission are certainly key elements to success. This consistency approach, when approved and in place, will allow new and established products of assured quality to reach the patient in a more efficient manner.

Science, newly developed methods and relevant guidelines are available to support the move to a Consistency Testing - based - control strategy. Adapting the mindset was one of the major hurdles to a progressive vision but there is now a consensus between experts from industry and regulatory bodies to move toward more aggressively eliminating *in vivo* testing.

### Definitions

***Release assay*.** A release assay is an assay, which is used to assess the quality of a given batch of the final product before the release on the market, or a production intermediate prior to its entry into further manufacturing steps. Its result must be within approved pre-defined acceptance criteria.

***Characterization assay*.** Physicochemical, immunochemical or biological assay which is used to accumulate knowledge on the product (final product or production intermediate) that will contribute to the overall product understanding. A characterization assay is deployed specifically in the context of evaluating the impact of process changes on the product.

***Critical Quality Attributes (CQA)*.** A physical, chemical, biological or microbiological property or characteristic that has been identified to be related to the product’s safety or efficacy and that should be within an appropriate limit, range, or distribution to ensure the desired product quality.

***Critical Process Parameter (CPP)*.** A process parameter whose variability has an impact on a critical quality attribute and therefore should be monitored or controlled to ensure the process yields a product of the desired quality.

***Acceptance Criteria*.** Numerical limits, ranges, or other suitable measures for acceptance of the results of analytical procedures, which the drug substance or drug product, intermediates or materials at defined stages of manufacturing process should meet.

## Ethics and consent

Ethical approval and consent were not required.

## Data Availability

No data are associated with this article.
