# Supplementary material for: The consistency approach for the substitution of in vivo testing for the quality control of established vaccines: practical considerations and progressive vision
Source: Open Res Eur. 2022 Dec 13;2:116. Originally published 2022 Oct 5. [Version 2] doi: 10.12688/openreseurope.15077.2 (PMC10446066; doi:10.12688/openreseurope.15077.2)
Supplement: Peer review history

# Peer review: Reviewer response for version 2

**DOI:** 10.21956/openreseurope.16629.r30480

**Published:** 2023-01-05

**Authors:** Elliot Lilley

**Affiliations:** 1 NC3Rs, London, UK

I am happy with the revised manuscript.

Where applicable, are recommendations and next steps explained clearly for others to follow? (Please consider whether others in the research community would be able to implement guidelines or recommendations and/or constructively engage in the debate)

Yes

Does the article adequately reference differing views and opinions?

Yes

Are all factual statements correct, and are statements and arguments made adequately supported by citations?

Yes

Is the rationale for the Open Letter provided in sufficient detail? (Please consider whether existing challenges in the field are outlined clearly and whether the purpose of the letter is explained)

Yes

Is the Open Letter written in accessible language? (Please consider whether all subject-specific terms, concepts and abbreviations are explained)

Partly

Reviewer Expertise:

I am Programme Manager for the 3Rs in vaccine batch release and quality control testing at the UK National Centre for the 3Rs (NC3Rs). After a 15-year career in the pharmaceutical industry, I spent 9 years with the RSPCA research animals department where I worked to promote refinement. I joined the NC3Rs in 2020 to work on a project to apply the 3Rs to batch testing of biologics, working closely with the WHO and a working group of international experts. I am a Fellow of the British Pharmacological Society and animal ethics editor of the British Journal of Pharmacology.

I confirm that I have read this submission and believe that I have an appropriate level of expertise to confirm that it is of an acceptable scientific standard.

# Peer review: Reviewer response for version 1

**DOI:** 10.21956/openreseurope.16305.r30255

**Published:** 2022-10-21

**Authors:** Kirsty Reid, Laura Viviani

**Affiliations:** 1 European Federation of Pharmaceutical Industry and Association, Brussels, Belgium; 2 Humane Society International, Geneva, Switzerland

It was a privilege to review a letter on such an important topic. We have some general comments for further consideration:

- Throughout the text in the abstract, summary, additional key elements, conclusion - there is a mix of use of words manufactures vs industry, regulatory bodies vs competent authorities. Just ensure consistency and wording used is that which best describes the stakeholder referenced.
- Is there a need to elaborate briefly on what is IMI?
- In the first paragraph of the Introduction section, I recommend including the link to the IMI webpage on the topic also: https://www.imi.europa.eu/projects-results/project-factsheets/vac2vac
- In the last paragraph of the Introduction could you consider referencing the following publications as they provide, not only the state of the art of the developing of in vitro opportunities to vaccines batch release testing, but also share the discussion, engagement and consensus level of various type of stakeholders on the topic. The manuscript mentions the shift of mindset that happened in the last years, and those publications reported such shift so they strengthen the points made in the letter.

1. Accelerating Global Deletion of the Abnormal Toxicity Test for vaccines and biologicals. Planning common next steps. A workshop Report: https://www.sciencedirect.com/science/article/pii/S1045105622000422 ^1^
2. Animal testing for vaccines. Implementing replacement, reduction and refinement: challenges and priorities: https://www.sciencedirect.com/science/article/pii/S1045105620300907 ^2^
3. Modern science for better quality control of medicinal products “Towards global harmonization of 3Rs in biologicals”: The report of an EPAA workshop:
https://www.sciencedirect.com/science/article/pii/S1045105617300647

- In the text there is a need for a final check of spacings, duplication of words (e.g. p5 'that are' is repeated in paragraph 6).
- P5 - for consideration in the text. An example below is covid specific but the concept of regulatory alignment, harmonization and resilience is well described. The same concept is key in the case of the substitution of in vivo methods. Lack of regulatory alignment is one of the major hurdles. Similarly, the consistency approach concept might incur in resistance in its acceptance and implementation.
  Mic McGoldrick et al. How to accelerate the supply of vaccines to all populations worldwide? Part I: Initial industry lessons learned and practical overarching proposals leveraging the COVID-19 situation, Vaccine, Volume 40, Issue 9, 2022, https://doi.org/10.1016/j.vaccine.2021.11.098.) ^2^
- Suggestion for the repeated use of the phrase 'Consistency Testing SPACE hyphen SPACE based SPACE hyphen control strategy'. Here it seems that “based” is a separate word, while, it means the Consistency Testing-based control strategy. Consider use of this suggested structure. Furthermore for the same phrase, sometimes the words all start with an upper case, others only certain words are upper case. Decide on one approach and keep it consistent.
- Abbreviations: First time European Directorate for the quality of Medicines and HealthCare is used, include the abbreviation (EDQM) next to it (p6). OCABR is mentioned, however never included in full in text - add *Official Control Authority Batch Release* to p7 before the abbreviation.
- P6 in the first paragraph under the heading 'stability studies' there is the reference to ' *removal of the in vivo*' consider also including the word ' *substitution or removal'*. 
- On p7 Paragraph starting ' *However, the comparison of results....*' is very confusing. It refers to 2 methods, then 2 procedures, then 2 quantitative methods. Please try to clarify for lay person.
- On p7 paragraph starting ' *Modern analytical technologies..*.'  please consider that the in vivo method might fail the requirement of the ICH Q2(R2) criteria for validation
- p8 when a paragraph is quoted from the PhEur, this should be in italics.
- Final sentence of final paragraph of the Conclusion, the word ' *aggressive*' is used. This is an odd choice of words. Reconsider using a different term here.
- In addition to the suggested publications above, I would recommend strengthening the letter by considering other publications which have come through the VAC2VAC projects: https://europevaccine.wixsite.com/vac2vac-eu/publications

Where applicable, are recommendations and next steps explained clearly for others to follow? (Please consider whether others in the research community would be able to implement guidelines or recommendations and/or constructively engage in the debate)

Yes

Does the article adequately reference differing views and opinions?

Yes

Are all factual statements correct, and are statements and arguments made adequately supported by citations?

Yes

Is the rationale for the Open Letter provided in sufficient detail? (Please consider whether existing challenges in the field are outlined clearly and whether the purpose of the letter is explained)

Yes

Is the Open Letter written in accessible language? (Please consider whether all subject-specific terms, concepts and abbreviations are explained)

Yes

Reviewer Expertise:

Animal research, animal welfare, alternatives, IMI, IHI, biology

We confirm that we have read this submission and believe that we have an appropriate level of expertise to confirm that it is of an acceptable scientific standard.

## References

[1] : Accelerating Global Deletion of the Abnormal Toxicity Test for vaccines and biologicals. Planning common next steps. A workshop Report. *Biologicals* .2022;78: 10.1016/j.biologicals.2022.06.003 17-26 10.1016/j.biologicals.2022.06.003 35840492PMC9490484

[2] : Animal testing for vaccines. Implementing replacement, reduction and refinement: challenges and priorities. *Biologicals* .2020;68: 10.1016/j.biologicals.2020.07.010 92-107 10.1016/j.biologicals.2020.07.010 PMC754378733041187

[3] : Modern science for better quality control of medicinal products "Towards global harmonization of 3Rs in biologicals": The report of an EPAA workshop. *Biologicals* .2017;48: 10.1016/j.biologicals.2017.05.006 55-65 10.1016/j.biologicals.2017.05.006 28596049

[4] : How to accelerate the supply of vaccines to all populations worldwide? Part I: Initial industry lessons learned and practical overarching proposals leveraging the COVID-19 situation. *Vaccine* .2022;40(9) : 10.1016/j.vaccine.2021.11.098 1215-1222 10.1016/j.vaccine.2021.11.098 PMC884626035180993

# Response

**Published:** 2022-11-22

**Authors:** Jean-Francois Dierick

**Affiliations:** GSK Vaccines, Belgium

Thank you very much for your constructive comments. We have addapted our publication accordingly and believe this leads to an improved version. We'd like to provide you our responses to the following comments:

- Is there a need to elaborate briefly on what is IMI? 

We prefer to keep our text focused on the main purpose of the publication. Several links included in the manuscript are directing towards websites that are referring to IMI.

- P5 - for consideration in the text. An example below is covid specific but the concept of regulatory alignment, harmonization and resilience is well described. The same concept is key in the case of the substitution of in vivo methods. Lack of regulatory alignment is one of the major hurdles. Similarly, the consistency approach concept might incur in resistance in its acceptance and implementation.
  Mic McGoldrick et al. How to accelerate the supply of vaccines to all populations worldwide? Part I: Initial industry lessons learned and practical overarching proposals leveraging the COVID-19 situation, Vaccine, Volume 40, Issue 9, 2022, https://doi.org/10.1016/j.vaccine.2021.11.098.) ^2^

The authors consider that this publication is not necessary for the reader to understand the manuscript and prefer not to add this reference that is realted to the very specific case of Covid vaccines and is not focused on 3Rs objectives.    

- On p7 Paragraph starting ' *However, the comparison of results....*' is very confusing. It refers to 2 methods, then 2 procedures, then 2 quantitative methods. Please try to clarify for lay person

We agree" with your comment, however, the confusion is coming from the original text from Ph.Eur. 5.2.14 that uses both “method” and “procedure”. We have therefore proposed not to change the text.

- On p7 paragraph starting ' *Modern analytical technologies..*.'  please consider that the in vivo method might fail the requirement of the ICH Q2(R2) criteria for validation

We are not confident in writing that “the in vivo method might fail the requirement of the ICH Q2(R2) criteria for validation” since this could be misinterpreted as the if the current control strategy was not appropriate. And this is not the case. We understand your comment and believe its essence is covered by the bullet point above the one discussed: “In vivo methods present a high level of variability that do not allow proper, statistically- relevant comparison of methods based on a reasonable amount of data (and animals).”

- In addition to the suggested publications above, I would recommend strengthening the letter by considering other publications which have come through the VAC2VAC projects: https://europevaccine.wixsite.com/vac2vac-eu/publications

The VAC2VAC consortium has indeed produced several very interesting publications in the context of the replacement of in vivo assays like, for instance the development of alternative in vitro assays. However, these publications are not illustrating or supporting the deployment of the consistency approach per se. We therefore propose not to add these references that risk to blur the specific message of this manuscript We sincerely hope that the revised version together with our responses to your comments will meet your expectations.

# Peer review: Reviewer response for version 1

**DOI:** 10.21956/openreseurope.16305.r30253

**Published:** 2022-10-14

**Authors:** Elliot Lilley

**Affiliations:** 1 NC3Rs, London, UK

Overall, this is a very important letter and worthy of publication. The authors have summarised the output from two workshops, organised by the VAC2VAC consortium, to discuss how manufacturers, control laboratories and regulators can work together to promote the use of the “Consistency Approach” to avoid the use of animals in quality control, batch and lot-release testing of vaccines. The letter sets out clear recommendations and makes a valuable contribution to the promotion of the 3Rs in routine vaccine testing.

 Sometimes the language is a little over-complicated in places and there are a number of typographical and grammatical errors.

 For example, in the introduction the following sentence:

 'In that perspective, the initiative launched reflection on how to support the deployment of the “Consistency Approach” for quality control of established vaccines, where current quality control approaches are often relying on *in vivo* methods.' does not read well.

 I would suggest the following: 'As part of the project, an initiative was launched to canvas opinion on how to support the deployment of the “Consistency Approach” for quality control of established vaccines, where current approaches often rely on *in vivo* methods.

 I would suggest that the manuscript is revised to improve the written English. This would greatly help to clarify the important message that the authors wish to communicate.

Where applicable, are recommendations and next steps explained clearly for others to follow? (Please consider whether others in the research community would be able to implement guidelines or recommendations and/or constructively engage in the debate)

Yes

Does the article adequately reference differing views and opinions?

Yes

Are all factual statements correct, and are statements and arguments made adequately supported by citations?

Yes

Is the rationale for the Open Letter provided in sufficient detail? (Please consider whether existing challenges in the field are outlined clearly and whether the purpose of the letter is explained)

Yes

Is the Open Letter written in accessible language? (Please consider whether all subject-specific terms, concepts and abbreviations are explained)

Partly

Reviewer Expertise:

I am Programme Manager for the 3Rs in vaccine batch release and quality control testing at the UK National Centre for the 3Rs (NC3Rs). After a 15-year career in the pharmaceutical industry, I spent 9 years with the RSPCA research animals department where I worked to promote refinement. I joined the NC3Rs in 2020 to work on a project to apply the 3Rs to batch testing of biologics, working closely with the WHO and a working group of international experts.  I am a Fellow of the British Pharmacological Society and animal ethics editor of the British Journal of Pharmacology.

I confirm that I have read this submission and believe that I have an appropriate level of expertise to confirm that it is of an acceptable scientific standard, however I have significant reservations, as outlined above.

# Response

**Published:** 2022-11-22

**Authors:** Jean-Francois Dierick

**Affiliations:** GSK Vaccines, Belgium

Thank you for your constructive comments. The entire text has been reviewed by a native speaker and has been improved. We hope these improvement will meet your expectations.
